# Carbonic anhydrase XII is a marker of good prognosis in invasive breast carcinoma

**DOI:** 10.1038/sj.bjc.6600796

**Published:** 2003-04-01

**Authors:** P H Watson, S K Chia, C C Wykoff, C Han, R D Leek, W S Sly, K C Gatter, P Ratcliffe, A L Harris

**Affiliations:** 1 Department of Pathology, D212-770 Bannatyne Avenue, University of Manitoba, Winnipeg, Manitoba, Canada R3E OW3; 2 Division of Medical Oncology, British Columbia Cancer Agency, Vancouver, British Columbia, Canada V5Z 4E6; 3 Cancer Research UK, Molecular Oncology Laboratory, University of Oxford, Institute of Molecular Medicine, John Radcliffe Hospital, Oxford OX3 9DU, UK; 4 Edward A. Doisy Department of Biochemistry, St Louis University School of Medicine, St Louis, MO 63104, USA; 5 Nuffield Department of Clinical Laboratory Sciences, University of Oxford, John Radcliffe Hospital, Oxford OX5 9DU, UK; 6 Wellcome Trust Centre for Human Genetics, Oxford OX3 7BN, UK

**Keywords:** carbonic anhydrase, breast cancer, hypoxia, marker, prognosis

## Abstract

Hypoxia and pH influence gene expression in tumours, and it is becoming increasingly clear that the pattern of genes expressed by a tumour determines its growth and survival characteristics. Hypoxia-inducible factor-1 (HIF-1) is a key mediator of the cellular response to hypoxia and high HIF-1 expression has been identified as a poor prognostic factor in tumours. Recently, we identified the tumour-associated carbonic anhydrases (CA), *CA9* and *CA12* as hypoxia-inducible in tumour cell lines. Furthermore, we identified CA IX to be a poor prognostic factor in breast cancer. The aim of this study was to assess the prognostic significance of CA XII. CA XII expression was studied by immunohistochemistry in a series of 103 cases of invasive breast cancer and any association with recognised prognostic factors or relation with the outcome was examined. CA XII expression was present in 77 out of 103 (75%) cases and was associated with lower grade (*P*=0.001), positive estrogen receptor status (*P*<0.001), and negative epidermal growth factor receptor status (*P*<0.001). Furthermore, although CA XII expression was associated with an absence of necrosis (*P*<0.001), expression of CA XII in some high-grade tumours was induced in regions directly adjacent to morphological necrosis. Additionally, using univariate analysis, CA XII positive tumours were associated with a lower relapse rate (*P*=0.04) and a better overall survival (*P*=0.01). In conclusion, CA XII expression is influenced both by factors related to differentiation and hypoxia in breast cancer *in vivo* and CA XII expression is associated with a better prognosis in an unselected series of invasive breast carcinoma patients.

Methods for the detection of breast cancer have improved markedly over the past decade and patients are subsequently presenting with ever earlier stages of disease (Ernster and Barclay, 1997). Although established markers of tumour behaviour, including tumour size and nodal status are applicable for this group of patients, tissue-based markers that can predict the risk of progression and recurrence would improve the ability to identify those who would benefit from the treatment (Bennington *et al*, 1992; Ernster *et al*, 1996; Tabar *et al*, 2000). Indeed, several such markers, such as tumour grade and estrogen receptor (ER) status are well established. These factors mostly relate to cellular differentiation, while alternative indicators of the metabolic status of tumour cells, such as oxygenation, are relatively sparse (Brizel *et al*, 1997; Vaupel and Hoeckel, 1999). Necrosis is one such indicator of poor oxygenation and has been shown to be a prognostic indicator (Leek *et al*, 1999). However, necrosis is nonspecific, and may not always be associated with severe hypoxia (Mueller-Klieser *et al*, 1983; Parliament *et al*, 1997; Ramanujan *et al*, 2000). To identify endogenous hypoxic markers that might have utility as prognostic indicators, we and others have begun to examine the expression patterns of known hypoxia-inducible genes in tumour specimens. Potential markers include the oxygen-regulated subunits of the transcriptional complex hypoxia-inducible factor-1 (HIF-1), a key mediator of the hypoxic response, HIF-1*α* and HIF-2*α*, or HIF-1 target genes (Shweiki *et al*, 1992; Maxwell *et al*, 1997; Zhong *et al*, 1999).

Recently, we extended the range of HIF-1 target genes by identifying the two tumour-associated transmembrane carbonic anhydrases (CA) *CA9* (Opavsky *et al*, 1996; Ivanov *et al*, 1998) and *CA12* (Ivanov *et al*, 1998; Tureci *et al*, 1998) as upregulated by hypoxia in a range of epithelial cancer cell lines (Wykoff *et al*, 2000). Carbonic anhydrases catalyse the reversible hydration of carbon dioxide to carbonic acid, providing a potential link between metabolism and pH regulation. We were therefore interested in examining the expression of these CA in tumourigenesis and progression in breast cancer. Our initial studies focused on CA IX as this gene demonstrated a more dramatic hypoxic induction in the tumour cell lines examined (Wykoff *et al*, 2000). CA IX expression was closely correlated with the presence of necrosis in both *in situ* and invasive cancer (Chia *et al*, 2001; Wykoff *et al*, 2001), and was found to be an independent predictor of relapse-free and overall survival (Chia *et al*, 2001). In the present study, we have investigated CA XII expression in breast cancer in the anticipation that its expression might also serve as an indicator of tissue hypoxia and tumour progression. Specifically, we wished to assess the pattern of expression in invasive breast carcinoma and the relation to outcome.

## MATERIALS AND METHODS

### Patients and tissues

A series of 103 surgically resected invasive breast carcinomas was assessed for CA XII expression in sections from paraffin tissue blocks. The clinicopathologic characteristics of this cohort are representative of that of a general population of breast cancer patients and are summarised in Table 1

**Table 1:** Clinical and pathological characteristics

**Patient characteristics**	**Number**
Total patients	103
*Age (median, range)* (years)	59 (28–83) years
<50	27
>50	76
	
*Surgical treatment*	
Lumpectomy	5
Lumpectomy+RT	63
Mastectomy	35
	
*Tumor size (median, range)* (cm)	2.4 cm (0.8–8 cm)
<2	43
>2	58
	
*Node status*	
Positive (number positive nodes)	58 (1–16)
Negative	45
	
*Grade*	
1	15
2	48
3	40
	
*ER status*	
Positive	70
Negative	33
	
*EGFR status*	
Positive	56
Negative	46
	
*Adjuvant therapy*	
Chemotherapy	27
Hormonal therapy (Tamoxifen)	80
	
*Median duration follow-up (median, range)*	6.2 (0.4–10.1)
Relapses	41
Deaths	32

. The distribution of tumour types was 87% invasive ductal, 11% invasive lobular, and 2% special type (mucinous and tubular). All cases had previously been treated at the John Radcliffe Hospital and the Churchill Hospital, Oxford, and all cases underwent either modified radical mastectomy or lumpectomy with breast irradiation for primary treatment. Axillary lymph node positive cases also received adjuvant radiotherapy to the axillary region. Adjuvant systemic treatment consisted of endocrine therapy (for all postmenopausal women regardless of hormonal receptor status and comprising Tamoxifen at 20 mg daily for 5 years) or chemotherapy (comprising six cycles of intravenous cyclophosphamide, methotrexate and 5-fluorouracil delivered every 3 weeks). All patients were assessed by follow-up every 3 months for the first 18 months and then every 6 months thereafter. Treatment for confirmed recurrent disease was by endocrine therapy for soft tissue or skeletal metastasis or by chemotherapy for visceral disease and failed endocrine therapy.

### Assessment of tumour grade, necrosis, and hormone receptor status

Tumour grade and necrosis was scored on haematoxylin- and eosin-stained sections immediately adjacent to those sections subjected to immunohistochemistry. Tumour grade was assessed using the Nottingham system (Elston and Ellis, 1991). The entire section was also assessed for the percentage of necrosis present within the invasive tumour component. Both parameters were scored by a single pathologist (PHW) independently from the IHC analysis and blinded to the cohort's clinical data and outcome. Necrosis was assessed semiquantitatively by estimating the area of the tumour section involved by light microscopy. Necrosis within *in situ* carcinoma components was not scored. For statistical analysis the percentage of necrosis was either assessed as a continuous variable or divided into negative or positive (where the presence of any necrosis was considered positive). Estrogen receptor (ER) analysis was performed using an enzyme linked immunosorbent assay technique (Abbott Laboratories, Chicago, USA). Epidermal growth factor receptor (EGFR) was determined using ligand binding of [^125^I] EGF to tumour membranes (Fox *et al*, 1994). Tumours with an EGFR level greater than or equal to 20 fmol mg^−1^of membrane protein and ER greater than or equal to 10 fmol mg^–1^ of cytosol protein were considered positive.

### Immunohistochemistry (IHC)

Immunohistochemical staining for CA XII was performed on 5 *μ*m serial sections on coated slides from paraffin-embedded blocks. The anti-CA XII antibody (Kivela *et al*, 2000a; Tureci *et al*, 1998) has previously been characterised and we have also further confirmed its ability to specifically detect CA XII expression in tissue sections by correlation with Western blot analysis in human breast tumour specimens (Wykoff *et al*, 2001). All slides were first deparaffinised and then placed in 0.5% hydrogen peroxide for 15 min to saturate endogenous peroxidases. Incubation with 10% normal human serum in Tris buffered saline (TBS) for 15 min was then performed to block nonspecific uptake of the antibody. The anti-CA XII antibody (Kivela *et al*, 2000a) was used at a dilution of 1 : 50 in TBS with 5% normal human serum for 30 min. Next, a 30 min incubation with a peroxidase conjugated to goat anti-mouse immunoglobulins (Dako EnVision+System, Peroxidase, Mouse; Dako, Carpinteria, CA, USA) was performed. Slides were then stained with 3,3-diaminobenzidine chromogen solution for 8 min and then counterstained with haematoxylin and mounted with aquamount. All staining was performed on an automated immunohistochemical stainer (MiniPrep 75, Tecan; Reading, UK) at room temperature (RT). Following successive incubations (except the normal human serum block) the slides were washed twice with TBS for 5 min.

### Assessment of CA XII expression

Immunostaining for CA XII was quantified by light microscopy and semiquantitative scoring by one author (PHW) blinded to the cohort's clinical data and outcome. In brief, a score of 0–3 was assigned for the intensity of staining assessed at low-power magnification (× 10 objective; 0: no staining; 1: weak staining; 2: moderate staining; and 3: strong staining). The percentage of invasive tumour cells that were stained positive was then estimated at both low and high magnification and the product of the intensity of staining and the percentage of tumour positive cells was then calculated to produce an immunostaining score (IHC score) of 0–300.

### Statistical analysis

For statistical analysis CA XII expression was evaluated as a continuous variable using the IHC-score. CA XII was also assessed as a categorical variable using the median (IHC score >40) to distinguish low from high expression. CA XII was evaluated in relation to a range of established prognostic variables using Mann–Whitney and Kruskal–Wallis tests where appropriate. The association with relapse-free and overall survival was assessed by univariate (log-rank test and Kaplan–Meier method) and multivariate (Cox's regression model) analysis.

## RESULTS

### CA XII expression in relation to clinicopathological variables

**Figure 1:**
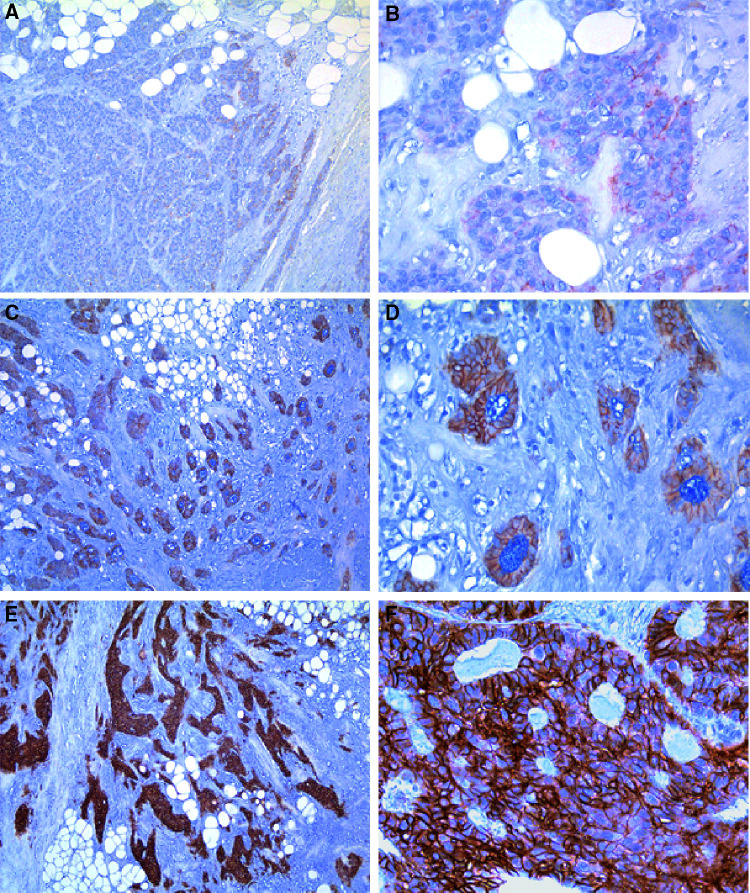
Expression of CA XII in invasive breast tumours. The panels show representative examples of CA XII expression detected by IHC in three invasive ductal carcinomas. The cases were associated with overall IHC scores of 10 (**A** and **B**), 100 (**C** and **D**), and 225 (**E** and **F**) respectively. Panels on the right side (**B**, **D** and **F**) are higher magnifications to show cellular detail within the corresponding tumour shown on the left. Original magnifications; left × 40, right × 200.

The specificity of the rabbit anti-human CA XII antisera for detection of CA XII in breast tissue sections was initially confirmed by comparison of IHC scores with the detection of 46–48 kDa bands corresponding to CA XII by immunoblotting performed on the same tumour specimens (Wykoff *et al*, 2001). CA XII expression was observed in 77 out of 103 (75%) cases of invasive carcinoma with a wide distribution of IHC scores (range 0–270, median 40, mean 65). Immunostaining was restricted to membranous staining of epithelial tumour cells and the pattern of expression was usually homogeneous. Representative examples of tumours showing overall low, moderate and high CA XII expression are illustrated in Figure 1

.

**Figure 2:**
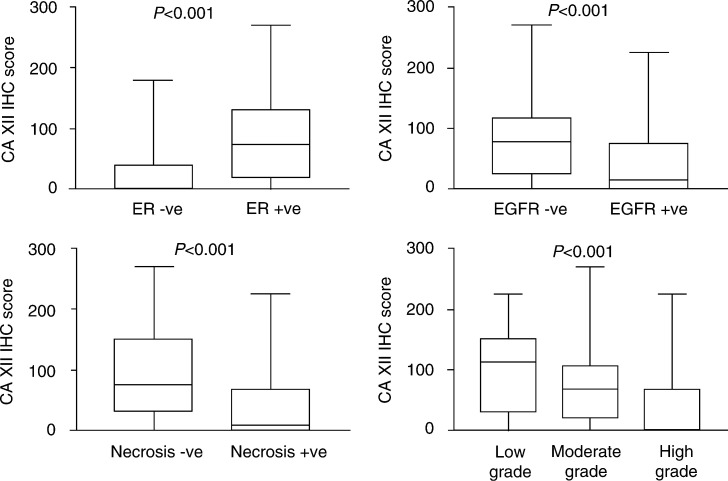
Association of CA XII with ER, EGFR, necrosis and grade. Each box indicates the range of CA XII IHC score from the 25th percentile to the 75th percentile, the line indicates the median, and the whiskers above and below the box show the highest and lowest values.

CA XII expression was compared with several established clinical–pathological prognostic variables. When CA XII was assessed as a continuous variable, there was a strong correlation between CA XII expression and tumour grade (*r*=−0.39, *P*<0.0001 Spearman's test), ER level (*r*=0.53, *P*<0.0001), EGFR (*r*=−0.38, *P*<0.0001) and extent of necrosis (*r*=−0.44, *P*<0.0001). When CA XII was assessed as a categorical variable these relations were also evident, with high CA XII expression significantly associated with lower tumour grade, positive ER status, negative EGFR status, and the absence of necrosis (Figure 2

). Despite the negative association between overall CA XII staining and necrosis, accentuation of CA XII expression was sometimes observed in tumour cells immediately adjacent to areas of necrosis within both *in situ* and invasive components (Figure 1), as might be predicted from our previous *in vitro* studies with tissue-culture cells (Wykoff *et al*, 2000). However, this express-ion pattern was typically present only focally in high-grade tumours where the overall level of CA XII within the tumour was low. No other significant relation with other prognostic variables such as age, nodal status, tumour size, or tumour type were observed.

### Relation of CA XII expression to relapse-free survival and overall survival

**Figure 3:**
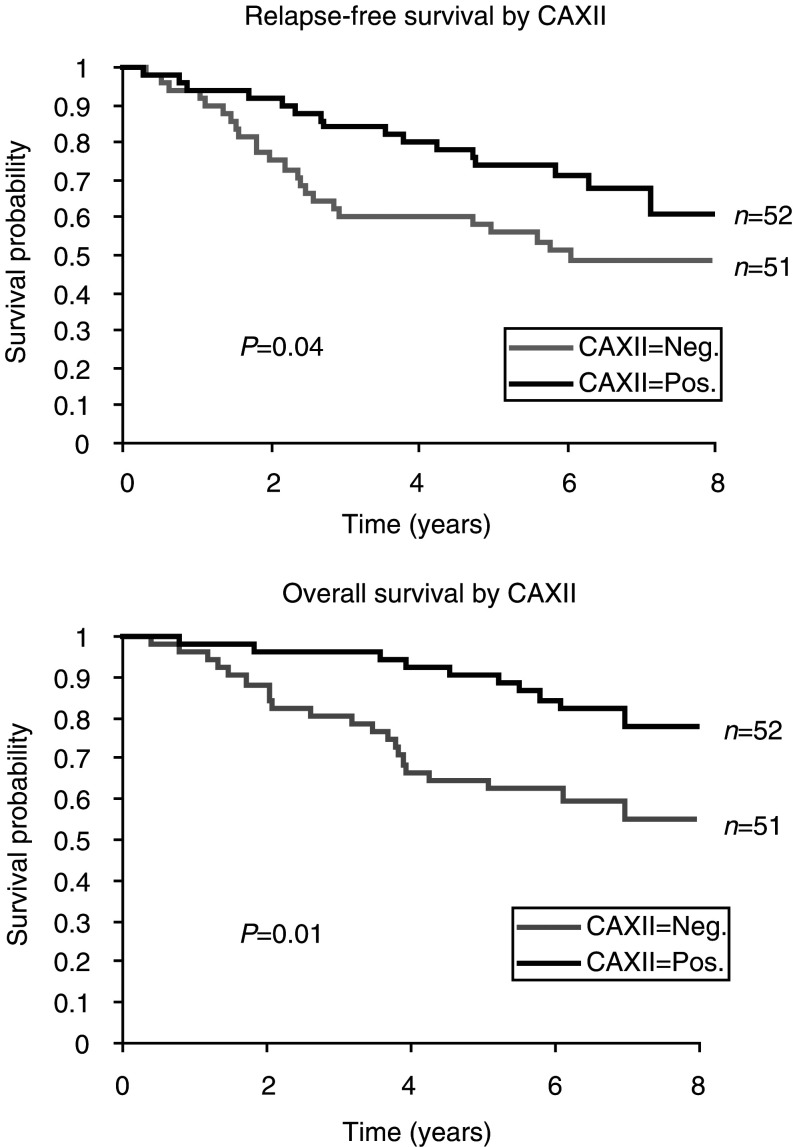
Kaplan – Meier curves demonstrating a relation between CA XII and relapse-free and overall survival within the entire cohort.

**Figure 4:**
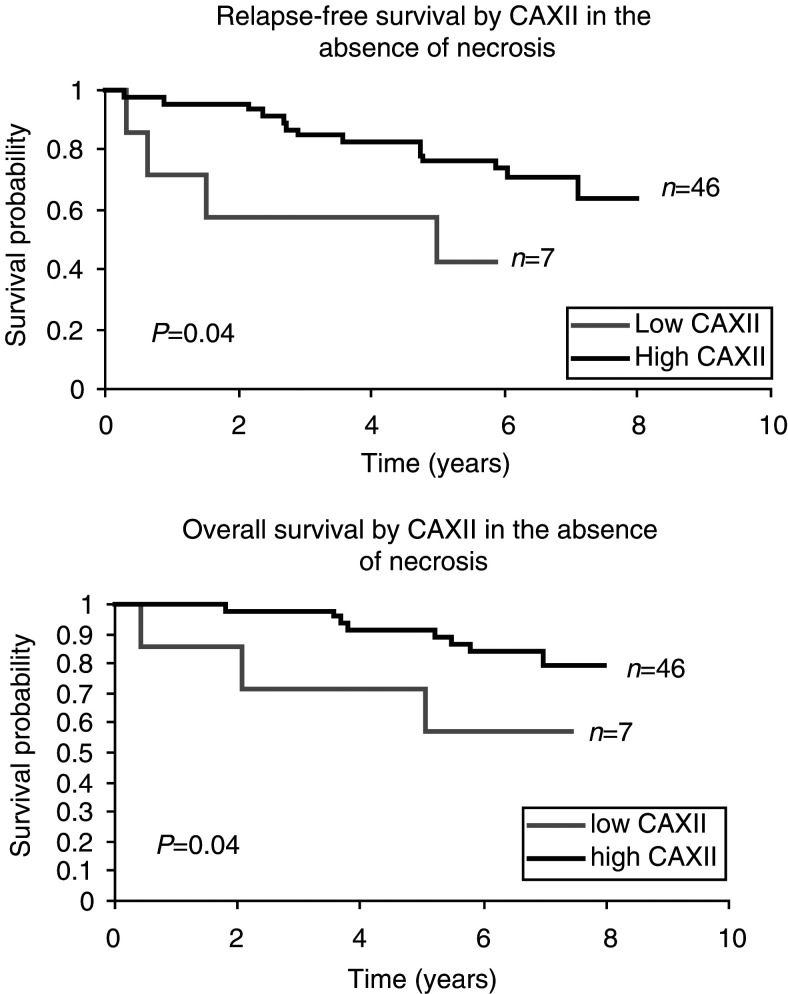
Kaplan – Meier curves demonstrating an association of CA XII with outcome within subgroups of tumours with and without necrosis.

Univariate analysis was conducted to explore the relation of CA XII to established prognostic factors and to survival. The relation of several conventional factors with survival in this cohort of cases was first confirmed. Nodal status, grade, and ER status were all significantly related to both relapse-free survival (*P*-values=<0.001, 0.04, 0.03, respectively) and overall survival (*P*-values=<0.001, 0.04, 0.02, respectively) in this series. In addition, tumour size (relapse-free survival, *P*=0.04) and patient age (overall survival, *P*=0.05) were also predictive of aspects of survival. Further analysis, considering CA XII expression as a categorical value (IHC score 0, CA XII negative; IHC score >0, CA XII positive), showed a significant association between CA XII positive tumours and a longer relapse-free survival (*P*=0.04) and a better overall survival (*P*=0.01) (Figure 3

). In addition, subgroup analysis showed that the presence of CA XII expression was also a significant predictor of both longer relapse-free and better overall survival for tumours without necrosis (*P*=0.04, 0.04, Figure 4

).

We were unable to demonstrate an independent influence in multivariate Cox's proportional hazard analysis when CA XII, lymph node status, grade, ER status and necrosis were all considered in the model. However it should be noted that amongst these factors, only lymph node status emerged as an independent predictor of survival in this cohort of patients.

## DISCUSSION

In this study, we have shown that CA XII is frequently expressed in invasive breast carcinoma, where expression is strongly associated with several good prognostic parameters, including low tumour grade, ER-positive status, EGFR-negative status, and absence of necrosis.

These findings extend our previous observations that expression of CA XII is closely related to tumour differentiation in pre-invasive *in situ* ductal breast carcinomas (DCIS), where CA XII was observed to be highest in well differentiated and reduced in poorly differentiated lesions (Wykoff *et al*, 2001). Further-more, although CA XII expression was generally low or absent in high-grade tumours with necrosis, we observed that focal induction of CA XII can be present in tumour cells adjacent to necrosis. This is consistent with our previous observations in *in situ* breast carcinoma (Wykoff *et al*, 2001) and similar observations made by others in invasive breast carcinoma (Ivanov *et al*, 2001), and with our *in vitro* findings that CA XII is regulated to some degree by hypoxia in breast tumour cells (Wykoff *et al*, 2000).

CA XII is widely expressed in specialised cells within normal tissues (Ivanov *et al*, 2001) and in several different tumour types (Tureci *et al*, 1998; Kivela *et al*, 2000a, 2000b; Ivanov *et al*, 2001). In one previous study of 29 breast cancers, CAXII was found to be focally expressed in normal breast and expressed in 62% of invasive carcinomas, primarily in low-grade tumours (Ivanov *et al*, 2001). In the normal colon and associated cancers, CA XII expression was frequently found in superficial regions of normal colonic mucosal epithelium, and was found to persist at comparable levels within preinvasive and invasive lesions (Kivela *et al*, 2000a). Although no significant association was seen with tumour grade, it should be noted that relatively small subgroups were available for analysis of invasive carcinoma and that the cranial–caudal location of each lesion may also have influenced expression. Despite this, a trend was observed towards reduced expression in high- *vs* low-grade adenoma, complementing our findings in preinvasive breast cancer (Wykoff *et al*, 2001). In contrast to this pattern of decreasing expression with increasing grade, Kivela *et al* (2000a) observed increased expression in nonsurface, deep cryptal epithelium with progression from normal through *in situ* to invasive disease, between low- and high-grade adenomas, and in higher stage invasive tumours. It is interesting to speculate that both these regional differences and the increased expression of CA XII in deeper regions within adenomas and colon carcinomas might relate to differences in vascularity, oxygenation and relative hypoxia.

Although other members of the CA gene family have been examined in several tumour types (Nogradi, 1998), and it is known that the increased expression of CA IX is associated with aspects of early tumourigenesis in cervix, colon and lung tumours (Liao and Stanbridge, 1996; Saarnio *et al*, 1998; Vermylen *et al*, 1999), a prognostic role for CA has not previously been identified in breast cancer. Our recent studies have shown that increased CA IX expression is associated with poor relapse free and overall survival in invasive breast cancer (Chia *et al*, 2001). CA IX expression is also closely related to necrosis. Necrosis may be attributable to several factors (Mueller-Klieser *et al*, 1983; Parliament *et al*, 1997; Ramanujan *et al*, 2000), but an important factor is likely to be hypoxia and the relation between CA IX and necrosis is consistent with the recently described relation between HIF-1*α* and prognosis in tumours (Zhong *et al*, 1999). However, the results of the current study indicate that while hypoxia may influence CA XII expression in focal areas discernable within high-grade breast tumours, the regulation of CA XII by differentiation-related factors appears to be dominant *in vivo*. Consequently, CA XII is associated with several phenotypic characteristics consistent with well differentiated tumours and good overall survival, at least in univariate analysis. The relatively small size of the series limited the capacity to study CA XII and prognosis within subsets of tumours with otherwise good prognosis. However, CA XII appears also to be a discriminator of good outcome within tumours that are negative for necrosis. Our results suggest that further examination of the role of CA XII as a prognostic marker may be warranted within certain subgroups of patients. The possibility that CA XII expression may also be relevant to chemotherapy response in such cases should also be considered, since an acidic extracellular pH can reduce uptake and efficacy of doxorubicin (Raghunand *et al*, 1999).

In conclusion, we have shown that CA XII is frequently expressed in invasive breast carcinoma and that focal enhancement of expression can be observed adjacent to areas of necrosis, supporting the notion that CA XII is a hypoxia regulated gene *in vivo*. However, the dominant factors in CA XII regulat-ion are clearly related to tumour differentiation and higher levels of CA XII expression are associated well with differentiated tumours and with a better relapse-free and overall survival. In view of the known differences in extracellular pH (Gerweck, 1998) and the contrasting patterns of expression observed for CA IX and XII in normal and neoplastic breast tissues (Wykoff *et al*, 2001), it will be important to explore the prognostic significance of CA XII further, in concert with other carbonic anhydrases and in relation to specific chemotherapies that may be influenced by extracellular pH (Raghunand *et al*, 1999), in larger prospective studies.
